# Chicken serum uric acid level is regulated by glucose transporter 9

**DOI:** 10.5713/ajas.20.0092

**Published:** 2020-06-24

**Authors:** Xuedong Ding, Chenglu Peng, Siting Li, Manman Li, Xinlu Li, Zhi Wang, Yu Li, Xichun Wang, Jinchun Li, Jinjie Wu

**Affiliations:** 1 College of Animal Science and Technology, Anhui Agricultural University, Hefei 230036, China

**Keywords:** Chicken, Layer, Uric Acid, Glucose Transporter 9

## Abstract

**Objective:**

Glucose transporter 9 (GLUT9) is a uric acid transporter that is associated with uric absorption in mice and humans; but it is unknown whether GLUT9 involves in chicken uric acid regulation. This experiment aimed to investigate the chicken GLUT9 expression and serum uric acid (SUA) level.

**Methods:**

Sixty chickens were divided into 4 groups (n = 15): a control group (NC); a sulfonamide-treated group (SD) supplemented with sulfamonomethoxine sodium via drinking water (8 mg/L); a fishmeal group (FM) supplemented with 16% fishmeal in diet; and a uric acid-injection group (IU), where uric acid (250 mg/kg) was intraperitoneally injected once a day. The serum was collected weekly to detect the SUA level. Liver, kidney, jejunum, and ileum tissues were collected to detect the GLUT9 mRNA and protein expression.

**Results:**

The results showed in the SD and IU groups, the SUA level increased and GLUT9 expression increased in the liver, but decreased in the kidney, jejunum, and ileum. In the FM group, the SUA level decreased slightly and GLUT9 expression increased in the kidney, but decreased in the liver, jejunum, and ileum. Correlation analysis revealed that liver GLUT9 expression correlated positively, and renal GLUT9 expression correlated negatively with the SUA level.

**Conclusion:**

These results demonstrate that there may be a feedback regulation of GLUT9 in the chicken liver and kidney to maintain the SUA balance; however, the underlying mechanism needs to be investigated in future studies.

## INTRODUCTION

Uric acid (UA) is the final product of exogenous and endogenous purine metabolism, and its over-generation or under-excretion may lead to hyperuricemia [1]. Normally, serum uric acid (SUA) levels are balanced between liver production and renal and intestinal excretion [2]. Approximately 90% of the SUA is filtered in the glomerulus, and then reabsorbed in the renal tubules. However, UA is a polar molecule and, thus, cannot freely pass through the cell membrane. Therefore, the process of UA reabsorption depends on ion channels. A genome-wide association study has revealed numerous genes (encoding for UA transporters) that are associated with hyperuricemia and gout [3].

Glucose transporter 9 (GLUT9) was originally thought to a glucose transport. However, a previous study showed that the inactivation of glucose transporter 2 in the liver completely blocked glucose uptake by cells and GLUT9 was still highly expressed, indicating that the main role of GLUT9 is not glucose transportation [4]. Later, Vitart et al [5] used Xenopus oocytes to express splice variants of the human solute carrier family 2 member 9 (*SLC2A9*) gene and discovered that human GLUT9 (hGLUT9) is a transport protein for UA. Subsequently, some studies have demonstrated that hGLUT9 is closely related to UA metabolism [6,7], it is a high-capacity/high-affinity UA transporter, and it is the only member of the GLUT family that transports UA [8,9]. The rate of UA transport is 45- to 60-fold greater than that of glucose. Therefore, GLUT9 may be a common transporter for both glucose and UA. Furthermore, in mouse, GLUT9 expression is also related to the SUA level, and high GLUT9 expression may cause hyperuricemia by increasing UA reabsorption in mouse kidneys [6]. Research has shown that GLUT9 is mainly expressed in the liver, kidney and the intestine of mice and humans [10,11], and it plays a very important role in UA regulation in mice and humans. In mice, liver GLUT9 helps absorb blood urea into hepatocytes for uricase-mediated degradation; renal tubule GLUT9 helps reabsorb UA from the urine back into the blood circulation [12]. Thus, mouse GLUT9 (mGLUT9) plays a major role in UA homeostasis through its dual functions in the kidney and liver [13, 14]. However, human liver lacks uricase [15] and therefore GLUT9 expression may differ from that in mouse. Current research on GLUT9 mainly involves human and mouse kidney UA-transport systems [16,17], whereas relevant studies in the human liver and intestine are limited. Due to the lack of uricase, chicken UA metabolism may be similar to that in humans; therefore, chicken may be an ideal model for functional studies of the human UA transporter.

A phylogenetic analysis of the chicken GLUT9 (cGLUT9) amino acid sequence revealed that it is identical to that of the *hGLUT9* gene, and that GLUT9 is expressed in the liver of chicken, is related to glucose metabolism, and is regulated by insulin [18,19]. However, substrate specificity of cGLUT9 remains unknown. Studies of whether cGLUT9 is involved in regulating chicken UA levels have not been conducted. Some research suggest that high-protein diets [20], sulfamonomethoxine [21], and intraperitoneal UA injections can increase the SUA level [22]. This study aims to identify the expression of cGLUT9 in the liver, kidney, jejunum and ileum of the chicken, and determine its relationship with the SUA level.

## MATERIALS AND METHODS

### Experimental design

In this experiment, sixty female ISA Brown laying hen chicks (twenty days old) were randomly divided into four groups (n = 15 per group). The control group (NC) was fed the basal diet; the sulfonamide group (SD) was fed the basal diet, with soluble sulfamonomethoxine sodium powder added to the drinking water (8 mg/L). Hengxin Pharmaceutical, Shijiazhuang, China); the fishmeal group (FM) was fed the basal diet supplemented with 16% fishmeal (crude protein, 25.6%); and the UA-injected group (IU) was fed the basal diet and administered UA (250 mg/kg, Sigma, St. Louis, MO, USA) by intraperitoneal injection (UA suspended in 0.5% carboxymethyl cellulose-Na solution) (Solarbio, Shanghai, China) once a day. The experiment lasted three weeks, and all the chickens were reared in cages and allowed *ad libitum* consumption of feed and water. The room temperature was 25°C to 30°C and 12 h light. The diet compositions were based on the recommended requirements of the National Research Council (1994) (Table 1). After fasting for 12 h, the chickens blood samples were collected from their jugular vein once a week; the blood was allowed to clot for approximately 30 min at 37°C, and then centrifuged at 3,500×*g* for 10 min to obtain the serum. The collected serum was stored at −20°C. At the end of the experiment (41 days of age), six chickens from each group were euthanized by decapitation. Liver, kidney, jejunum, and ileum tissues were collected and stored for the subsequent analyses. This study protocol was approved by the Animal Care and Ethics Committee of Anhui Agricultural University (ZXD-P2017625).

### Determination of the serum uric acid Level

The SUA level was measured (UA enzymatic method) using an automated biochemical analyzer [23] (BS-220; Mindray, Shenzhen, China) according to the manufacturer’s instructions.

### Detection of GLUT9 mRNA expression by quantitative real-time polymerase chain reaction

Total RNA from the liver, kidney, jejunum and ileum (100 mg) was extracted based on a previously published study [24]. Tissues were ground in liquid nitrogen and homogenized in 1 mL of Trizol (Thermo Scientific, Waltham, MA, USA). The extracted RNA concentrations were determined on a NanoVue Plus instrument (Thermo Scientific, USA). Then, reverse transcription of 500 ng of total RNA was performed, followed by quantitative polymerase chain reaction (qPCR) at 95°C 2 min, 40 cycles at 60°C for 1 min, 60°C for 30 s, 60 to 95°C in 0.2°C/s, and 10 s at 20°C. The sequences of the primers used for the qPCR analysis of chicken GLUT9 (SLC2A9) expression are shown in Table 2. The experiment was replicated three times. The relative expression of mRNA was analyzed using the 2^−ΔΔCt^ method [25].

### Detection of GLUT9 protein expression by western blot

Total protein was extracted from the liver, kidney, jejunum and ileum (100 mg) of fowl using 1 mL of RIPA cell lysate (Biosharp, Shanghai, China), 10 μL of protease inhibitor (Solarbio, China), and 20 μL of phosphatase inhibitor (Solarbio, China), and the resulting mixture was homogenized for 2 min. The cells were lysed at 4°C for 20 min. The lysates were then centrifuged at 12,000×g for 10 min at 4°C. The supernatant was aspirated and stored at −80°C. The protein concentration was determined using the BCA method [26] (Biosharp, China).

For western blotting, 50 μg of the treated sample was elec trophoresed at 80 V for 40 min on a 5% sodium dodecyl sulfate (SDS) -polyacrylamide stacking gel, followed by electrophoresis at 120 V for 80 min on a 10% SDS-polyacrylamide running gel. The proteins were electrophoretically transferred on to a polyvinylidene fluoride membrane at 120 V for 26 min. The rabbit anti-GLUT9 antibody (diluted 1:1,000; Novus, Littleton, CO, USA) and a goat anti-rabbit immunoglobulin G (IgG) secondary antibody (diluted 1:5,000; ImmunoWay, Plano, TX, USA) were used. The proteins were visualized using a Western Blot Detection Kit (Advansta, Menlo Park, CA, USA). The density of the bands was analyzed using the Image Pro Plus software version 6.0 [27] (Media Cybernetics, Washington, MD, USA), and the protein expression was normalized to β-actin band.

### Detection of GLUT9 protein expression by immunohistochemistry

The tissues (liver, kidney, jejunum, and ileum) were fixed in 4% paraformaldehyde, paraffin-embedded, and cut into 5 μm thick sections. Next, the sections were deparaffinized and dehydrated, and then rinsed three times with phosphate-buffered saline (PBS). Subsequently, 3% hydrogen peroxide was added dropwise to the sections for 25 min to block the endogenous peroxidase activity. The sections were blocked with bovine serum albumin (5%) at room temperature for 25 min in the dark and incubated overnight at 4°C with diluted anti-GLUT9 polyclonal antibody (1:400; Novus, USA), as described by Liu et al [28]. After 12 h, the sections were washed three times with PBS, and then a goat anti-rabbit IgG secondary antibody (diluted 1:5,000; ImmunoWay, USA) was added dropwise to it. After incubation at 37°C for 30 min, the sections were washed again three times with PBS. Coloration was performed with DAB (Zsgb Biotechnology, Beijing, China) for three min, and then the sections were washed with distilled water, stained with hematoxylin, dehydrated, and mounted with a neutral gum. Finally, the immunolabeled sections were observed under a light microscope (Olympus CX31, Tokyo, Japan) and photographs were recorded. Integrated optical density (IOD) for each section was measured using the Image-Pro Plus version 6.0 [27] (Media Cybernetics, USA).

### Statistical analysis

Data are expressed as mean±standard error. Differences in SUA as well as GLUT9 levels between treatment groups and NC group were analyzed by one-way analysis of variance using LSD and Duncan’s multiple comparison post test using IBM SPSS Statistics [29]. Statistical significance is defined when p values are less than 0.05. The correlation analysis was conducted to explore the relationship between SUA and GLUT9 mRNA expression by Pearson’s correlation coefficients. Graphs and scatter plots were generated using GraphPad Prism [30].

## RESULTS

### Serum uric acid level of chickens

As shown in Table 3, on day 7, the SUA level was significantly increased (p<0.05) in the SD and IU groups compared with that in the NC group. On day 14, the SUA level was higher in the SD and IU groups than in the NC group, but not statistically significant. On day 21, the SUA level was increased (p = 0.119) in the SD group and significantly increased (p< 0.05) in the IU group compared with the NC group. The SUA level in the FM group was decreased throughout the experimental period compared with the NC group, but there was no significant difference compared with the NC group.

### GLUT9 mRNA and protein expression in the liver, kidney, jejunum, and ileum of normal chickens

**Figure 1:**
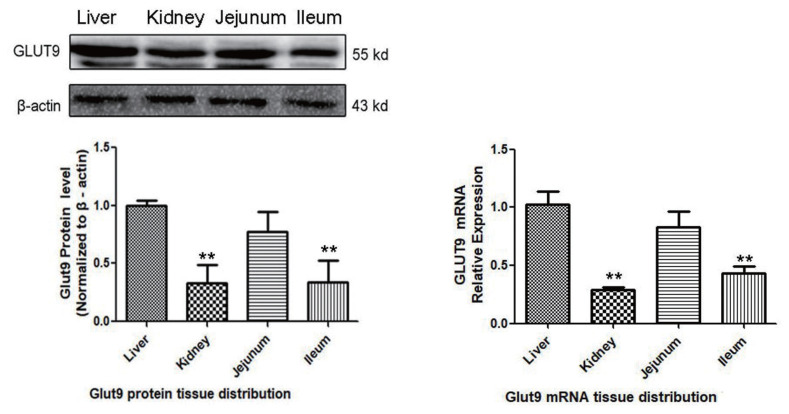
GLUT9 mRNA and protein expression in the liver, kidney, jejunum and ileum of normal chickens, as determined by qPCR and western blot (n = 6). The GLUT9 mRNA and protein expression levels in the kidney, jejunum and ileum were compared with those in the liver. GLUT9, glucose transporter 9; qPCR, quantitative polymerase chain reaction; NC, control group; SD, sulfonamide group; FM, fishmeal group; IU, uric-injected group. ** Indicates p< 0.01 compared with the NC group.

The mRNA and protein expression of GLUT9 was quantified in four major tissues of chicken. As shown in Figure 1, the GLUT9 mRNA and protein in normal chickens is mainly expressed in the liver, followed by the jejunum, then the ileum, and least in the kidney.

### GLUT9 mRNA and protein expression in the liver, kidney, jejunum and ileum of chickens in each group by qPCR and western blot

**Figure 2:**
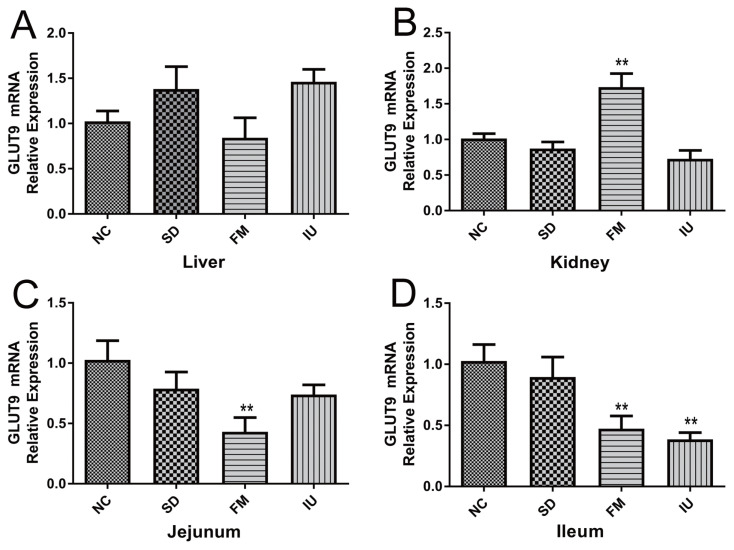
GLUT9 mRNA expression in the liver, kidney, jejunum and ileum of chickens in each group, as determined by qPCR (n = 6). GLUT9, glucose transporter 9; qPCR, quantitative polymerase chain reaction; NC, control group; SD, sulfonamide group; FM, fishmeal group; IU, uric-injected group. ** p<0.01 vs NC group (one-way analysis of variance).

The qPCR results were shown in Figure 2. Compared with the NC group, liver GLUT9 mRNA expression of the FM group was decreased, but increased in the SD and IU groups (Figure 2A). Conversely, renal GLUT9 mRNA expression was increased in the FM group (p<0.01), but decreased in the SD and IU groups (Figure 2B). The jejunum and ileum GLUT9 mRNA expression were decreased in the FM, SD, and IU groups (Figures 2C, D), compared with that in the NC group.

**Figure 3:**
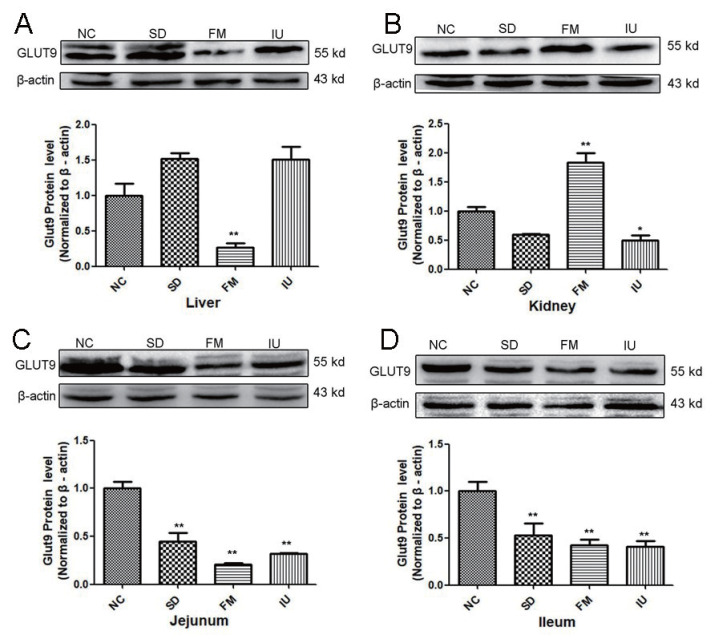
GLUT9 protein expression in the liver, kidney, jejunum and ileum of chickens in each group, as determined by western blot (n = 6). GLUT9, glucose transporter 9; NC, control group; SD, sulfonamide group; FM, fishmeal group; IU, uric-injected group. * p<0.05 and ** p<0.01 vs NC group (one-way analysis of variance).

The western blot results as shown in Figure 3, reiterated the qPCR results. Compared with the NC group, the liver GLUT9 protein expression was decreased in the FM group, but increased in the SD and IU groups (Figure 3A). On the other hand, renal GLUT9 protein expression was increased in the FM group, but decreased in the SD and IU groups (Figure 3B). The jejunum and ileum GLUT9 protein expression were decreased in the FM, SD, and IU groups (Figures 3C, D), compared with that in the NC group.

### GLUT9 protein expression in the liver, kidney, jejunum and ileum of chickens in each group by immunohistochemistry

**Figure 4:**
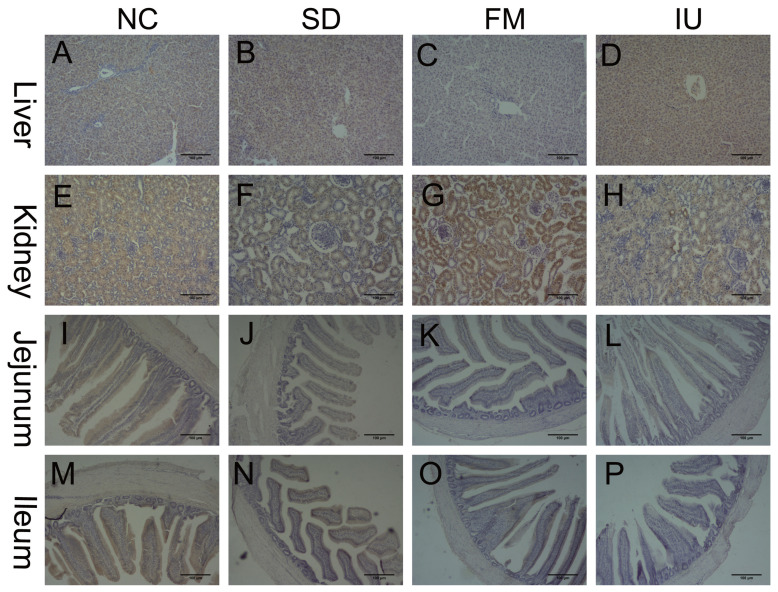
GLUT9 protein expression in the liver, kidney, jejunum and ileum of chickens in each group, as determined by immunohistochemistry (n = 6). Scale bars: 100 μm. GLUT9, glucose transporter 9; NC, control group; SD, sulfonamide group; FM, fishmeal group; IU, uric-injected group.

The immunohistochemistry results are shown in Figure 4 and Table 4. Compared with the NC group, liver GLUT9 protein expression was decreased in the FM group (p<0.01), but increased in the SD and IU groups. Conversely, renal GLUT9 protein expression was increased in the FM group (p<0.01), but decreased in the SD and IU groups. GLUT9 protein expression in the jejunum and ileum tissues was decreased in all the FM, SD, and IU groups, compared with that in the NC group (p<0.01).

### Correlation between the serum uric acid level and GLUT9 mRNA relative expression

**Figure 5:**
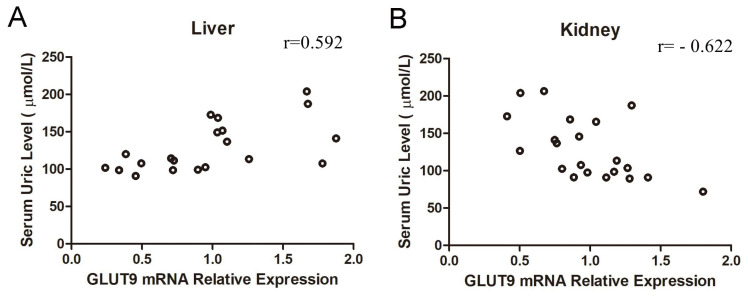
Correlation between the serum uric acid level and GLUT9 mRNA relative expression. GLUT9, glucose transporter 9.

Based on the results described above, a correlation analysis was performed to examine the relationship between the SUA level and GLUT9 expression in the liver and kidney. The results found that the SUA level was positively related to the GLUT9 mRNA expression in the liver (Figure 5A), with a correlation coefficient of 0.592. However, in the kidney, the SUA level was negatively correlated with GLUT9 mRNA expression (Figure 5B), with a correlation coefficient of −0.622. Furthermore, there were no obvious correlation between the SUA level and GLUT9 mRNA expression in the jejunum and ileum.

## DISCUSSION

The SUA level reflects the balance between UA production and excretion. The production of UA depends on the intake of dietary protein and the breakdown of endogenous purines by xanthine oxidase. The kidneys are the major site of UA excretion and the small intestines are the secondary sites [31]. Excessive UA production or low excretion can increase the SUA levels, leading to hyperuricemia. Approximately 90% of hyperuricemia is associated with reduced UA excretion and only 10% is caused by increased UA production [32]. In the present study, intraperitoneal UA injection was used to increase the SUA level, which is consistent with the results of a previous study [22]. Supply of sulfamonomethoxine in drinking water also increased the SUA level, which may be caused by sulfonamide-dependent blocking of the renal tubules and reduced UA excretion [21]. A previous study demonstrated that high-protein diets can increase the SUA level in chicken [20]. In the present study, supplementary fishmeal in the diet was used to increase the dietary protein level, but it did not increase the chicken SUA level, thus, further study is needed in this regard.

Similar to the findings of a previous study in mice [11], the results of the present study show that the cGLUT9 mRNA and protein is mainly expressed in the liver, followed by the jejunum, whereas the ileum and the kidney show less expression. The immunohistochemistry results indicate that the pattern of GLUT9 protein expression in the chicken liver is consistent with that in the humans and the mice; that is, it is mainly expressed in hepatocytes. However, GLUT9 expression in chicken renal tubules differ from that in humans and mice. mGLUT9 is mainly expressed in basolateral membrane of the renal proximal tubules and is weakly expressed in the proximal tubules [12,14,33]. The human GLUT9 protein is mainly localized in the basolateral membrane of the proximal tubules [10,34]. The immunohistochemistry results of the present study demonstrate that the cGLUT9 protein is expressed both in the renal proximal tubules and distal tubules. The renal tubules are the major site of UA reabsorption, and the differential expression of GLUT9 may reflect differences in UA reabsorption between human, mouse and chicken. The chicken intestinal GLUT9 protein is abundantly expressed in the jejunum and ileum, and it is mainly located in the apical membrane of intestinal villus cells. In mice, immunofluorescence staining show that the intestinal GLUT9 protein is mainly located in the basolateral and apical membranes of jejunal and ileal villus cells [35]. Therefore, GLUT9 protein expression in the liver, kidney, jejunum and ileum exhibit some similarities and differences between chicken, human and mouse. However, little is known regarding the molecular mechanisms of GLUT9 function in diverse tissues, which merits further study.

In mice, there are two main ways to eliminate SUA: via uricase-dependent degradation in the liver and via secretion by the kidney and intestines, and both the routes require the involvement of GLUT9 [12]. Liver GLUT9 is the main transport protein that transports blood urea into hepatocytes. When liver GLUT9 is specifically inactivated, mice develop severe hyperuricemia. In the present study, the SUA level was increased in the SD and IU groups, which may be associated with the increase in liver GLUT9 expression and decrease in renal GLUT9 expression. In the FM group, the SUA level was decreased slightly, and GLUT9 expression was increased in the kidney and decreased in the liver. A similar result has been reported by Nagura et al [36], who found that the SUA level in 5/6 nephrectomized mice is increased slightly and renal GLUT9 gene expression is decreased. These findings suggest that there may be a complex regulatory action mode between GLUT9 expression and SUA level. The results of the present study show that liver GLUT9 mRNA and protein expression in chicken correlated positively with the SUA level; while renal GLUT9 mRNA and protein negatively correlate with the SUA level. Thus, we hypothesize that feedback regulation of GLUT9 maintains the SUA balance in the chicken liver and kidneys. When the SUA level was reduced, GLUT9 expression was decreased in the liver, which in turn decreased SUA reabsorption in the liver. Simultaneously, GLUT9 protein expression was increased in the kidneys and UA reabsorption was increased in the renal tubules to raise the SUA level. Accordingly, when the SUA level was elevated, liver GLUT9 expression was increased and renal expression was decreased to lower the SUA level.

In mice, most UA excretion occurs in the jejunum and il eum and intestinal GLUT9 functions to regulate UA clearance; hyperuricemia occurs in intestinal GLUT9-deficient mice [35]. However, in the present study, we found that GLUT9 mRNA and protein expression in the jejunum and ileum of chicken was not related with the changes in the SUA level, warranting further investigation into the mechanism of GLUT9 function in chicken intestines.

In summary, the liver GLUT9 expression correlated posi tively with the SUA level, while renal GLUT9 expression correlated negatively with the SUA level. The GLUT9 expression in the jejunum and ileum does not seem to be significantly associated with the SUA level. Thus, there may be a feedback regulation of GLUT9 in the chicken liver and kidney that maintains the SUA balance, but the underlying mechanism is still unknown and requires further studies.

## Figures and Tables

**Table 1:** Dietary ingredients and main nutritional composition

Items	
Ingredient (%)	
Corn	55
Bran	5
Soybean meal	20
Cotton wool	3
Corn protein flour	5
Soybean oil	1
DDGS	5
Calcium powder	1
Premix1)	5
Total	100
Main nutritional composition	
Crude protein (%)	18.15
Calcium (%)	1.21
Phosphorus (%)	0.51
Methionine (%)	0.32
Lysine (%)	0.76
Total energy (MJ/kg)	11.55

DDGS, dried distillers grains with solubles.

1) Premix (Hualuo 5% premix for layer during brood period, Zhongmu, China). Premix supplied per kilogram of diet: vitamin A (retinyl acetate) 48.2 to 99.8 mg, vitamin E (dl-α-tocopheryl acetate) ≥350 mg, vitamin B_1_ ≥50 mg, vitamin B6 ≥80 mg, nicotinamide ≥550 mg, pantothenic acid 280 mg, zinc 1,000 to 3,000 mg, copper 5 to 700 mg, manganese 1,000 to 3,000 mg, selenium 2 to 10 mg, total phosphorus ≥2.6%, folic acid ≥16 mg, vitamin D_3_ ≥5 to 90,000 IU, vitamin K_3_ 40 to 75 mg, vitamin B_2_ ≥150 mg, vitamin B_12_ ≥0.3 mg, biotin ≥1.8 mg, iron 1,600 to 15,000 mg, choline chloride ≥7,000 mg, iodine 6 to 100 mg, calcium 16% to 22%, sodium chloride 5% to 9%.

**Table 2:** Chicken GLUT9 (SLC2A9) primers sequences for quantitative polymerase chain reaction

Gene	Primer sequence (5′–3′)	Length (bp)	Accession No.
*GLUT9-F*	GCATCATTCTGCATTGGACC	119	XM_420789.5
*GLUT9-R*	AAGTTGGAGAGCCAGTTGAC		
*18S-F*	CGGCGACGACCCATTCGAAC	99	M_59389.1
*18S-R*	GAATCGAACCCTGATTCCCCGTC		

*GLUT9*, glucose transporter 9; SLC2A9, solute carrier family 2 member 9.

**Table 3:** Serum uric acid level (μmol/L) of chickens in each group (n = 10)

Groups1)	0 d	7 d	14 d	21 d
NC	111.6±9.3	125.7±8.6	123.3±3.6	115.7±7.3
SD	112.0±4.1	158.4±12.6*	136.9±11.5	141.7±19.1
FM	110.7±7	119.1±9.6	107.7±8.3	112.9±7.8
IU	114.3±9.1	160.8±7.2*	134.5±4.1	152.9±6.1*

1) NC, control group; SD, sulfonamide group; FM, fishmeal group; IU, uric-injected group.

* p<0.05 vs NC group (one-way analysis of variance).

**Table 4:** Average optical density values of glucose transporter 9 protein in the liver, kidney, jejunum and ileum of chickens in each group by immunohistochemistry (n = 6)

Groups1)	Liver	Kidney	Jejunum	Ileum
NC	0.046±0.0013	0.039±0.0009	0.057±0.0021	0.067±0.0025
SD	0.051±0.0052	0.034±0.0011	0.039±0.0022**	0.053±0.001**
FM	0.019±0.001**	0.058±0.0048**	0.018±0.0008**	0.023±0.0022**
IU	0.050±0.005	0.027±0.0035*	0.02±0.0009**	0.022±0.0013**

1) NC, control group; SD, sulfonamide group; FM, fishmeal group; IU, uric-injected group.

* p<0.05 and

** p<0.01 vs NC group (one-way analysis of variance).
